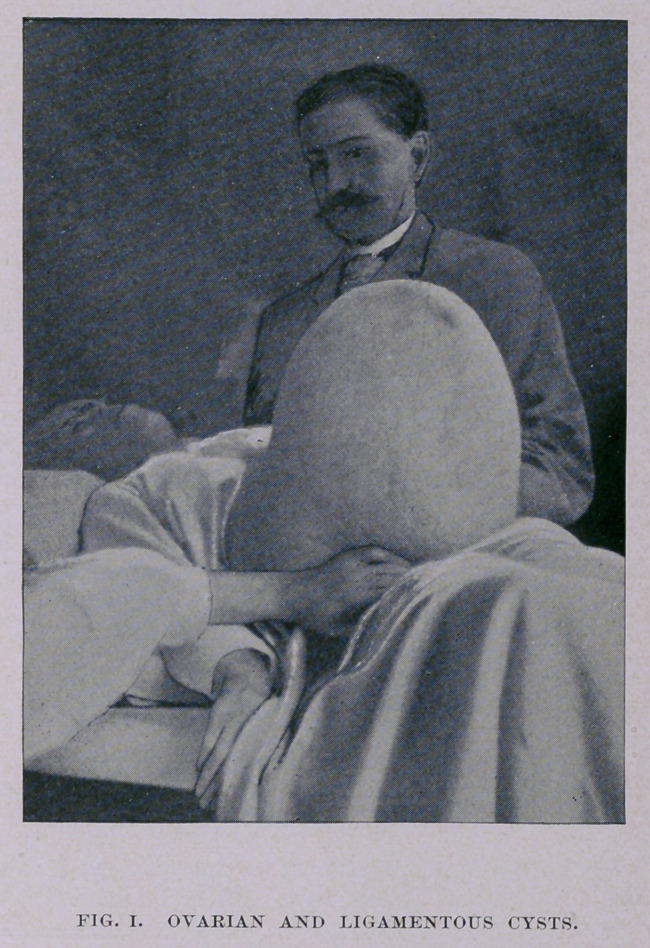# Ovarian and Ligamentous Cysts Co-existing in the Same Patient; Operation; Death from Shock1Read at the annual meeting of the American Association of Obstetricians and Gynecologists, in Philadelphia, September, 1890.

**Published:** 1891-03

**Authors:** William Warren Potter

**Affiliations:** Buffalo, N. Y., Consulting Gynecologist to the Maternity Hospital; Fellow of the American Association of Obstetricians and Gynecologists


### OVARIAN AND LIGAMENTOUS CYSTS CO-EXISTING IN THE SAME PATIENT; OPERATION; DEATH FROM SHOCK.[1]

By WILLIAM WARREN POTTER, M. D., Buffalo. N. Y.,

Consulting Gynecologist to the Maternity Hospital; Fellow of the American Association of Obstetricians and Gynecologists.

This case is presented for record by reason of the unusual shape and size of the patient's abdomen, the dual nature of the cysts, and as illustrating the dangers of repeated tappings and delay in submitting to an operation for radical cure:

Mrs. X., of Allegany county, New York, was referred to me by Dr. W. W. Crandall, presenting the following history: Age, twenty-six years, married; one child five years old, no miscarriages. Soon after confinement, more than five years ago, she observed that her abdomen began to enlarge, and within a few months she was tapped and fifteen quarts of fluid drawn. During the next three or four months she was tapped twice more, when ten and eight quarts of water were drawn at the respective tappings. During the past few years she has not been tapped, but her abdomen has become greatly distended, rendering it burdensome, and nearly or quite preventing locomotion. Her appetite and digestion were fairly good, menstruation regular, no constipation, kidneys moderately active, and bladder not irritable — all of which seemed remarkable when the size and shape of the belly were considered. She was greatly reduced in flesh, had a feeble heart, and presented other signs of failing vitality. There was a soft, protruding area to the left of the umbilicus, and slightly above its plane, which was strongly indicative of an approaching rupture of the cyst through the external abdominal walls.

Operation July 9, 1890. In making the abdominal incision it was noted that the usual anatomical landmarks were wanting, and the cyst-wall was slightly punctured unawares. It was with much difficulty that a start could be made in the separation of the cyst-wall from the peritoneum, and these were so stoutly adherent for so great an area that much time was required in freeing this cyst, which was without pedicle, and appeared to spring from the right broad ligament. Another small cyst was tied off which proved to involve the right ovary. On the left side was another large cyst made up of and including innumerable other cysts, varying from the size of a marble to an egg, besides a few nearly or quite the size of a Florida orange. The first or right cyst contained about five gallons of a brownish-yellow fluid mixed with flakes and masses of a soft, friable, fibrinous material. The left cyst contained, in

1. Read at the annual meeting of the American Association of Obstetricians and Gynecologists, in Philadelphia, September, 1890.

addition to the myriads of smaller ones, about three and a half gallons of fluid, ordinary in general appearance and characteristics. The ental walls of the right cyst were roughened, and in many places lined with a fibrinous material that formed thick deposits. The ectal walls were covered with shreds of connective tissue, especially ventrad where extensive adhesions had taken place between its ectal layers and the peritoneal surfaces.

The cysts had, in fact, two points of support: the one, ventrad, formed by the peritoneal adhesions; the other, dorsad, their point of departure from the uterine appendages. I have already mentioned the fusion between the cyst-walls and the peritoneum; this was so stout that at one time it was feared that it would thwart removal. There was, however, very little hemorrhage, in spite of all the difficulties presented.

This instance well illustrates the dangers that may surround a neglected case, and presents a strong argument for early operation. In justice to Dr. Crandall it is proper to add that, as soon as the patient consulted him, he advised her to make all possible haste in submitting herself to an operation, and he finally persuaded her to do so.

The woman took ether badly, and was despondent; moreover, she was devitalized by the long-continued strain and drain on her mental and physical powers. She died, from shock, within three or four hours after the completion of the operation.

The illustration (Fig. 1), from a photograph taken just before the operation, conveys a better impression of the size and shape of the tumor than any description can afford. The distorted abdomen looked, to use the graphically laconic words of Dr. Frederick, who was present and assisted me, as if "a huge watermelon had been implanted end-first in its walls."

### DISCUSSION.

Dr. ROBERT T. MORRIS, of New York.—As a rule, I am opposed to tapping ovarian cysts; however, in such cases as this, where the cyst is so large, it seems to me that we avoid the danger of shock if, one, two, or three days before the operation for its removal, we draw off a part of the fluid contents, the amount to be withdrawn to be determined by the judgment of the surgeon. All are familiar with the great shock that sometimes follows a simple tapping operation. If, then, we add to this the shock by removing the walls of the tumor, and disturb the large sympathetic plexuses of the abdomen, we paralyze the sympathetics which control the circulation of the blood, thus inhibiting digestion, causing the formation of gas, and great distress, to say nothing of the danger of disturbing the vaso-motors of the heart.

FIG. I. OVARIAN AND LIGAMENTOUS CYSTS.

Dr. POTTER (closing the discussion).—Perhaps I might have been a little more explicit in describing the case and the operation. It will be remembered that this patient was brought to me some distance from the country, and that she had already been tapped several times by some physician who apparently did not appreciate the importance of *always* withholding the trocar in this class of cases. On account of the great distention of the abdomen and the consequent visceral and vascular pressure, the propriety of an ante-section tapping was considered, and I discussed it with Dr. Frederick, now one of the Fellows of the Association, who was kind enough to assist me at the operation. We finally concluded that we would not further complicate the case, particularly in the patient's nervous state, by such a procedure, but that we would evacuate the fluid slowly during the operation, and make systematic and deliberate artificial pressure, with a view to counterpoise the sudden release of tension.

Dr. Morris has done wisely, in my view, in calling attention to the fact that ovarian cysts should not be subjected to early tapping. This is, I doubt not, the present attitude of abdominal surgeons on this question; there are not two opinions among men of experience in regard to it. To the early tappings, in the case I have reported, I attribute the extensive adhesions that so nearly baffled our efforts at removal of the right cyst.

The preliminary tapping of large cystomata that Dr. Morris advocates may be a very useful measure in properly selected cases, and it will be seen that it was considered, though rejected, in the present instance; but I believe it is an expedient that needs wise discrimination in its employment, and one of very limited application.

## Figures and Tables

**Fig. I. f1:**